# Evolutionary fates of universal stress protein paralogs in Platyhelminthes

**DOI:** 10.1186/s12862-018-1129-x

**Published:** 2018-02-01

**Authors:** Sergio Martin Espinola, Martin Pablo Cancela, Lauís Brisolara Corrêa, Arnaldo Zaha

**Affiliations:** 1 Programa de Pós Graduação em Genética e Biologia Molecular, Universidade Federal do Rio Grande do Sul, Porto Alegre, RS Brazil; 2 Centro de Biotecnologia, Universidade Federal do Rio Grande do Sul, Porto Alegre, RS Brazil; 3 Programa de Pós Graduação em Biologia Celular e Molecular, Universidade Federal do Rio Grande do Sul, Porto Alegre, RS Brazil

**Keywords:** Stress responsive proteins, Flatworms, Evolutionary patterns, Pseudogenes, Functional divergence

## Abstract

**Background:**

Universal stress proteins (USPs) are present in all domains of life. Their expression is upregulated in response to a large variety of stress conditions. The functional diversity found in this protein family, paired with the sequence degeneration of the characteristic ATP-binding motif, suggests a complex evolutionary pattern for the paralogous USP-encoding genes. In this work, we investigated the origin, genomic organization, expression patterns and evolutionary history of the USP gene family in species of the phylum Platyhelminthes.

**Results:**

Our data showed a cluster organization, a lineage-specific distribution, and the presence of several pseudogenes among the USP gene copies identified. The absence of a well conserved -*CCAATCA*- motif in the promoter region was positively correlated with low or null levels of gene expression, and with amino acid changes within the ligand binding motifs. Despite evidence of the pseudogenization of various USP genes, we detected an important functional divergence at several residues, mostly located near sites that are critical for ligand interaction.

**Conclusions:**

Our results provide a broad framework for the evolution of the USP gene family, based on the emergence of new paralogs that face very contrasting fates, including pseudogenization, subfunctionalization or neofunctionalization. This framework aims to explain the sequence and functional diversity of this gene family, providing a foundation for future studies in other taxa in which USPs occur.

**Electronic supplementary material:**

The online version of this article (10.1186/s12862-018-1129-x) contains supplementary material, which is available to authorized users.

## Background

The emergence of gene families is based on successive events of gene duplication. Duplicate copies can result from unequal crossing-over during meiosis or from retrotransposition processes [1]. While a crossing-over mismatch can generate a duplication of the entire gene structure, including promoter regions, introns and exons, retrotransposition events usually result in an intronless gene composed only of the exons of the ancestral gene, and giving rise to a single transcript. For each new duplicate copy, several outcomes are possible. First, a neofunctionalization process, where the new gene takes on a new function, different from that of the parental gene. Second, a subfunctionalization process, where the new copy preserves its function, but with a singular spatio-temporal regulation (e.g., expression in a specific tissue and at a specific developmental stage). Third, a pseudogenization process, where the duplicate copy accumulates deleterious mutations, leading to loss of function [2–4].

Members of the universal stress protein (USP) gene family are found in bacteria, archaea, and eukaryotes and are composed of a variable number of copies due to lineage-specific expansions [5]. These proteins are highly expressed in response to a large variety of stress conditions, such as oxidative stress, heat shock, and UV exposure [6–8]. In addition to stress resistance, they participate in the regulation of cell growth and host infection in *Mycobacterium tuberculosis* [9], and contribute to cell adhesion and motility in *Escherichia coli* [7]. The USP protein domain exhibits a protein motif capable to interact with ATP, ADP, AMP, GTP, etc. [9, 10]. In some USPs, the amino acid sequence making up this motif is partially or completely degenerated [11]. In general, it was observed that almost all USPs crystals with the typical ATP-binding motif were solved with ATP or an ATP analog, while for those USPs where this motif is completely degenerated, neither ligand nor ion binding was observed [11]. Although different functions have been identified for USPs with typical and degenerated ATP-binding motifs [6, 8, 12], the functional impact of the amino acid substitutions at sites involved in ligand interaction remains poorly understood.

The Platyhelminthes include some harmful parasite species with considerable negative effect on public health, especially in developing countries [13] (World Health Organization, WHO, 2016). Several of the so-called neglected tropical diseases (NTDs), a diverse group of communicable diseases of the tropics, are caused by Platyhelminthes, including echinococcosis and schistosomiasis, which are responsible for 1200 and 11,700 deaths per year worldwide, respectively [14]. The complex life cycle of parasitic Platyhelminthes involves their interaction with two or more hosts, accompanied by drastic physiological and morphological changes [15, 16]. This continuous change of microenvironments results in the exposure to a wide array of biotic and abiotic stressors [16–18]. In a recent review, the application of the USPs as novel anti-parasitic targets has been discussed [19]. USPs play an important role in the transition between different stages of the *Schistosoma* life cycle, including that between cercariae and schistosomula stages. Based on this and on the absence of this gene family in vertebrates, including humans, USPs could represent an interesting target for anti-schistosomal treatment [19].

Here, we use comparative genomics and the relationship between protein sequence variations and gene expression patterns to build a framework for the evolution of the USP gene family in the Platyhelminthes. This framework aims to explain the sequence and functional diversity of this gene family, providing a foundation for future studies in other taxa in which USPs occur.

## Methods

### Sample collection, genotyping and quantitative PCR

Bovine hydatid cysts were obtained from the Cooperleo Abattoir (São Leopoldo, Rio Grande do Sul, Brazil). The pre-adult stage (protoscoleces, PSC) of *Echinococcus ortleppi* was collected by hydatid cyst fluid aspiration and washed with phosphate buffered saline (PBS). Genotyping was performed on part of the cytochrome c oxidase subunit I (*cox1*) gene as previously described [20].

For quantitative PCR (qPCR) expression analysis, approximately 1.000 PSC were mixed with 0.5 mL of TRIzol reagent (Thermo Fisher Scientific) and immediately frozen in liquid nitrogen until RNA extraction. Total RNA was isolated using TRIzol according to the manufacturer’s protocol. Isolated RNA was subsequently treated with RNase-free DNase I (Sigma-Aldrich) for 30 min at 25 °C to remove all genomic DNA. Total RNA concentration was determined using a Qubit fluorometer (Thermo Fisher Scientific). The first strand of cDNA was synthesized from 200 ng of total RNA using M-MLV reverse transcriptase (Thermo Fischer Scientific) and an Oligo(dT)18 primer (0.5 μg/μL), following manufacturer’s instructions. The final cDNA product was diluted 100-fold with nuclease-free water prior to use in qPCR experiments.

Real-time PCR was performed using an ABI Real-Time 7500 Fast PCR system (Applied Biosystems). Based on the genome of *Echinococcus granulosus*, specific primers were designed for six USP genes of *E. ortleppi* (Additional file 1: Table S1), two of which are downregulated and four of which are upregulated in the pre-adult form, according to RNA-seq data [21, 22]. The reaction mixture and the qPCR cycling conditions were as described previously [23]. Control reactions without reverse transcriptase and without template were included to confirm the absence of genomic DNA and other PCR contaminants, respectively. All qPCR reactions were performed in technical and biological triplicates. The amplification efficiency was calculated using the LinRegPCR software [24]. The gene expression quantification was performed with the ΔΔCt method, using *EF-1α* as a normalizer gene [23].

### USP sequence retrieval

The USP sequences of twelve Platyhelminthes species (*Gyrodactylus salaris*, *Macrostomum lignano*, *Schistosoma mansoni*, *Schistosoma haematobium*, *Opisthorchis viverrini*, *Clonorchis sinensis, Echinococcus granulosus*, *Echinococcus multilocularis*, *Echinococcus canadensis*, *Taenia solium*, *Hymenolepis microstoma,* and *Schmidtea mediterranea*) were extracted from the WormBase ParaSite and SmedGD databases [25, 26] using the USP Pfam code PF00582 and the keyword “universal stress protein”. Orthologs relationship were initially obtained by reciprocal BLASTn, and confirmed by the presence of monophyletic clades of each group of the orthologs in the phylogenetic trees. USP sequences were retrieved based on the following criteria: synteny, the presence of a single exon in most sequences (or the conserved position of the intron where present) [5], and phylogenetic relationships between orthologs. The identification of low homology sequences (probable pseudogenes) was achieved through a tBLASTn search (BLOSUM45). For each species, we blasted each USP protein sequence against the entire genome, applying an e-value threshold of 1e-1, allowing the alignment of low complexity regions, and using opening and extending gap penalties of 14 and 2, respectively. To avoid the recovery of spurious hits, we used similar criteria to those used in the search for orthologs, as follows: synteny, genes with a single intron or intronless, and the amino acid conservation in specific regions related to the interaction with ligands and belonging to the USP domain. The USP sequences of the molluscs *Lottia gigantea*, *Crassostrea gigas*, and *Octopus bimaculoides*, and the annelids *Helobdella robusta* and *Capitella teleta*, were retrieved from the Ensembl Genome and JGI databases [27, 28] using the Pfam code PF00582. These last species were used as outgroups in the phylogenetic analysis.

### Phylogenetic trees

Phylogenetic analyses were performed using the Bayesian Inference (BI) and Maximum Likelihood (ML) probabilistic methods [29, 30]. Protein sequences were aligned with MAFFT v7 [31] using the FFT-NS-I method, and any columns containing more than 95% gaps were deleted using Gap Strip/Squeeze v2.1.0 [32]. The best substitution model for our data set was defined with the Smart Model Selection (SMS) tool incorporated in PhyML [33]. The ML tree was generated with PhyML v3.0 [29] using the aLRT-SH method for branch support. The BI tree was generated with BEAST v1.8.4 [30], using two independent runs of 50.000.000 chains and sampling at every 5.000 generations. The birth and death process [34], and the LG + G substitution model [35] with 4 gamma categories, were the priors for the analysis in BEAST v1.8.4. Other parameters (e.g. clock model) were used as default. The software TRACER v1.6 [36] was used to check the convergence of Monte Carlo Markov Chains (MCMC) and to ensure adequate effective sample sizes (ESS > 200) after the first 10% of generations were deleted as burn-in. The maximum clade credibility tree was estimated with TreeAnnotator, which is part of the BEAST v1.8.4 package, and the tree was visualized using Figtree v1.4.3 [37].

### Positive selection analysis

**Fig. 1:**
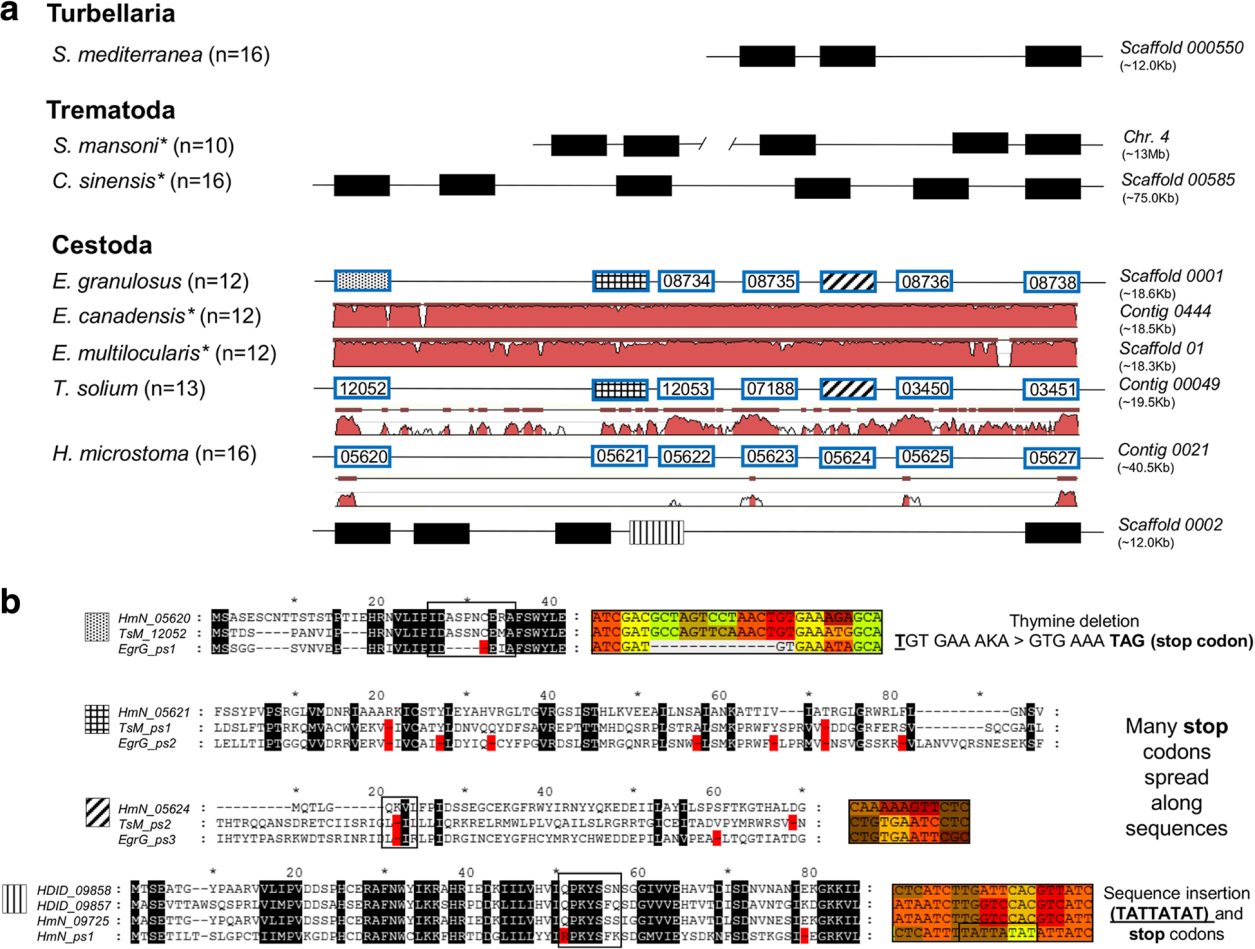
Organization of the USP gene family in Platyhelminthes. **a** Platyhelminthes USP genes are distributed in clusters throughout the genome and show lineage-specific expansions and losses. For each species, the total number of USP genes are given in parentheses, followed by a scheme (boxes) showing the genomic organization for some genes. Boxes with blue frame correspond to syntenic genes in Cestoda species. The sequence alignment of the entire cluster in Cestoda parasites (50% of identity at baseline and using *E. granulosus* as the reference sequence) display high levels of sequence identity in the coding region. Gene identity of USP genes is lost when compared to the Trematoda and Turbellaria classes, suggesting a high divergence of the USP genes between groups. Some USP paralogs in the Cestoda were identified as pseudogenes (dotted, gripped, and striped boxes). Because of the synteny, pseudogenes in *T. solium* and *Echinococcus* spp. correspond to gene losses when compared to *H. microstoma.* The *EgrG_ps1* pseudogene refers to the *EgrG_2019* sequence (WormBase Parasite annotation). Asterisks indicate the same *USP* distribution for *E. canadensis* and *E. multilocularis* compared to *E. granulosus*, and for *S. haematobium* and *O. viverrini* compared to *S. mansoni* and *C. sinensis*, respectively. **b** The presence of *indels* (highlighted in red in the protein sequence) generates frameshift mutations and serves as evidence of a pseudogenization process. The sequences of some pseudogenes (*EgrG_ps1*, *HmN_ps1*) are very similar to that of their orthologs. Others (*EgrG_ps2*) are very different, and homologies are difficult to identify accurately by tBLASTn. In addition to the protein alignment, the *indels* are indicated in the coding sequence for three pseudogenes

Positive selection was tested using the codeml program incorporated in the PAML package [38] and the mechanistic empirical combination model (MEC), as implemented in Selecton 7 [39, 40]. Due to the high divergence between the USP sequences of the different Platyhelminthes classes (see Fig. 1a), we limited the positive selection analysis to 63 coding sequences of the Cestoda class. Protein sequences were aligned with MAFFT v7 [31]. Subsequently, we use the program Pal2Nal [41] to align the coding sequences corresponding the protein alignment obtained with MAFFT v7. Considering codons (gaps in triplets), the ends of the sequences were removed manually, and columns containing more than 90% gaps were deleted using Gap Strip/Squeeze v2.1.0 [32]. The nonsynonymous/synonymous substitution rate ratio (*ω = d*_*N*_*/d*_*S*_) offers a sensitive measure of positively selected residues in proteins. Thus, a *ω* ratio greater than 1 suggest that nonsynonymous mutations would be adaptively advantageous in evolution and could be fixed in one or more populations. Thereby, lineages exposed to dissimilar pressures of selection on a given protein show differences in the *ω* ratio what could indicate these sites are on positive selection [42]. In order to verify whether the *ω* ratio deviated significantly from 1 for each alternative model considered, we performed a likelihood ratio test (LRT) on the results of the PAML run as follows: M1a (nearly neutral) versus M2a (positive selection); and M7 (beta) versus M8 (beta & *ω*).

### DNA motif analysis of the promoter regions

In order to gain insights about the origin and regulation of the USPs, we searched for conserved patterns (DNA motifs) in the promoter region of these genes. DNA motif analysis was performed using the mixture model by expectation maximization (MEME) method, incorporated in the MEME suite [43]. Five hundred bp of the USP promoter region were extracted from the 5′ end upstream of the start codon ATG. The *Saccharomyces cerevisiae* database was used to compare the identified motifs with others previously described (Tomtom) [44], and to find associations with genes linked to gene ontology terms (GOMo) [45]. The motif search was executed with default parameters, considering a maximum width of 10 nucleotides and allowing any number of repetitions for the motifs in the sequence. All upstream sequences are available in the Additional file 2.

### Divergence analysis and USP protein modeling

Evolutionarily conserved amino acids are expected to have an important role in protein structure and function. Therefore, changes at these sites may be an indicator of functional divergence. We used the software Diverge v3.0 [46], to examine site-specific shifted evolutionary rates by calculating the coefficient of type I of divergence (Ɵ_*I*_). Type I of divergence results in differing functional constraints (i.e., different evolutionary rates) between duplicated genes, regardless of the underlying evolutionary mechanisms. The null hypothesis (*Ɵ*_*I*_ = 0) is assessed by the likelihood ratio test (LRT) [47], and its rejection indicates some level of functional divergence between the clusters compared. Because the output of Diverge v3.0 follows a chi-square distribution with one degree of freedom, the LRT values greater than or equal to 3.84 indicate functional divergence between clusters. Comparisons were performed between paralogs groups of approximately five sequences within the Cestoda and Trematoda classes. Using a cut-off value of 0.9 for the a posteriori probability, we identified amino acid sites under Type I of functional divergence.

For protein modeling, we chose the *E. granulosus* USP protein EgrG_08736, which exhibits the typical ATP-binding motif [Gx2Gx9G(S/T)]. The 3D protein was modelled with a homologous template using Phyre v2 [48]. In addition to 3D modeling, Phyre v2 predicts ligand-binding sites and analyzes the effect of amino acid variants (Phyre Investigator). The obtained model was used to evaluate the effect of mutations at conserved sites, and to localize the amino acid residues found to be under functional divergence by Diverge v3.0. The quality of the model was assessed with ModFOLD v4.0 [49].

## Results

### USP gene organization in the Platyhelminthes

The high quality and completeness of several Platyhelminthes genomes (e.g. *Echinococcus* spp., *Schistosoma mansoni*) [21, 50], together with the criteria for the search for orthologs (see Methods), allowed us to locate and accurately retrieve all the DNA and protein sequences of the USP genes for each species. We found that the number of USP genes varied between Platyhelminthes species: 12 genes in *E. granulosus*, *E. multilocularis*, and *E. canadensis*, 13 in *T. solium*, 16 in *H. microstoma*, 10 in *S. mansoni* and *S. haematobium*, 18 in *C. sinensis* and *O. viverrini*, 17 in *S. mediterranea*, 6 in *G. salaris*, and 83 in *M. lignano* (Additional file 1: Table S2). The high number of USP sequences in this last species, including about 35 identical sequences, could be a consequence of an ancestral whole-genome duplication or recent large segmental duplications, as previously described [51]. To simplify, we removed the two zeros at the beginning and end of each USP identification number (ID) for the Cestoda species. Through reciprocal BLASTn, we detected orthologous relationships within the Cestoda and Trematoda classes; however, between classes, or when including the free-living flatworm *S. mediterranea* (class Turbellaria), the orthologous relationships between species become fuzzy and unrequited (Additional file 1: Table S2). In all Platyhelminthes species analyzed here, USP genes are distributed in clusters throughout the genome, with lineage-specific losses/expansions (Fig. 1a). Clustering is more accentuated in the Cestoda and Trematoda than in Turbellaria (data available at the SmedGD database). The relaxed tBLASTn analysis detected three pseudogene candidates in the genus *Echinococcus*, two in *T. solium*, and one in *H. microstoma*. Synteny indicates that these pseudogenes may represent lineage-specific gene losses in *Echinococcus* spp. and *T. solium* compared with *H. microstoma*; and in *H. microstoma* compared with *H. diminuta* (Fig. 1a and b). Pseudogenes are characterized by the presence of *indels* in their coding sequence, which lead to frameshift mutations and thereby generate stop codons (Fig. 1b). Sequence differences between pseudogenes and their respective ortholog are highly variable for paralogous pseudogenes, reflecting an ancient pseudogenization process (Fig. 1b). For the other species, there was no evidence of pseudogenes with our search strategy. Nevertheless, we identified twelve USP genes in the Trematoda, which could not be detected using the USP Pfam code. All of these were located in the vicinity of other USP genes. Two were not previously annotated (*Csin107892a*, *T265_02176a*), and one gene was re-annotated (*T265_02178*, corresponding to genes *T265_02178a* and *T265_02178b*). The other copies, which were annotated as “universal stress protein” or without description, were *Csin107891*, *Csin107892*, *Csin107893*, *Csin110039*, *Csin110041*, *T265_02177*, *T265_02179*, and *T265_02180*. All protein sequences reported in this work are available in the Additional file 2.

### Phylogenetic trees and origin of the USP gene family

**Fig. 2:**
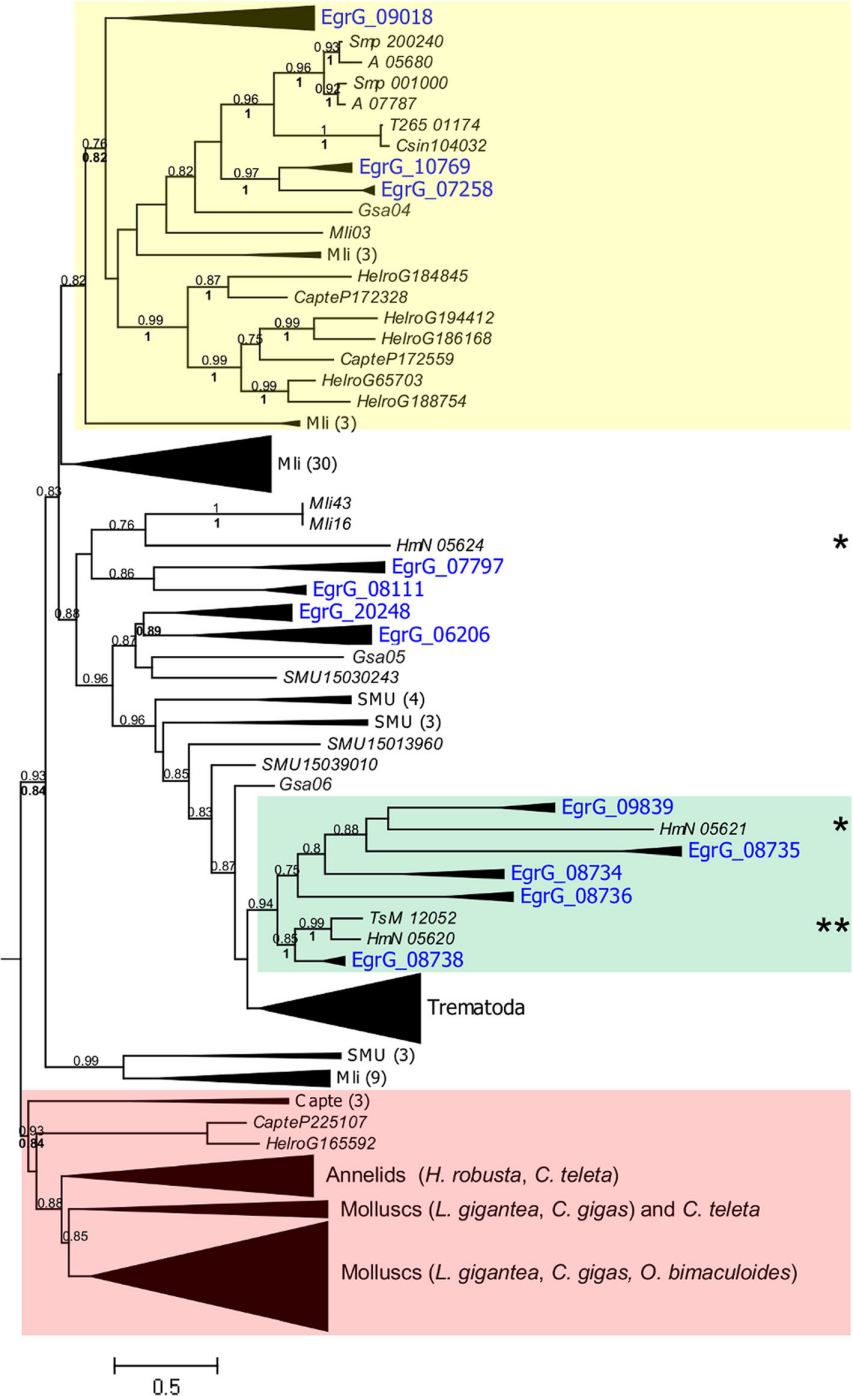
Phylogenetic relationships between USPs in the Platyhelminthes. The maximum-likelihood phylogenetic tree (method aLRT-SH for branch support, see text for more details) shows several USP sequences shared by platyhelminthes and annelids, referred as ancestral USP genes (highlighted in yellow). On the other hand, the Trematoda and Cestoda (highlighted in green) classes show species-specific expansions and losses (one asterisk indicates losses in the *Taeniidae* family; while two asterisks represent losses in the genus *Echinococcus*). For simplification (and to facilitate associations with Fig. 1), we grouped the Cestoda sequences according to the IDs of *E. granulosus* based on the ortholog relationship (see Additional file 1: Table S1). Gene names are in italic. Prefix species are as follow: CapteP for *C. teleta*, HelroG for *H. robusta*, Gsa for *G. salaris*, Mli for *M. lignano*, SMU for *S. mediterranea*, Smp for *S. mansoni*, Csin for *C. sinensis*, TsM for *T. solium*, HmN for *H. microstoma*, and EgrG for *E. granulosus*. The number in parenthesis beside SMU and Mli correspond to the number of collapsed sequences in *S. mediterranea* and *M. lignano*, respectively. The USPs clustered in the chromosome (see Fig. 1) are also grouped together in the phylogenetic tree, suggesting an origin by subsequent tandem duplications. Identical sequences from *M. lignano* (~ 35) were excluded in the analysis*.* Three molluscs (*L. gigantea*, *C. gigas*, and *O. bimaculoides*) and two annelids (*H. robusta* and *C. teleta*) were used as outgroups. A minor ID for *M. lignano* and *G. salaris* was used (Additional file 1: Table S5). For an extended tree, see Additional file 3: Figures S1 and S2. Branch support values obtained by Bayesian Inference are in bold font. Only values with a branch support greater than 0.7 are showed

Phylogenetic trees (Fig. 2; Additional file 3: Figures S1 and S2) showed five genes to be shared across all Platyhelminthes species analyzed here (gene names are given for *E. granulosus*): *EgrG_09018*, *EgrG_7258*, *EgrG_10769, EgrG_06206, EgrG_20248.* Of these, *EgrG_09018* and its orthologs are the only ones with a single intron. While a few sequences from annelids (*HelroG188754*, *HelroG65703*, *HelroG194412*, *HelroG186168*, *HelroG184845*, *CapteP172559*, and *CapteP172328*) were shared with Platyhelminthes (*EgrG_09018*, *EgrG_7258*, *EgrG_10769*), no homology was found for the molluscs species (Fig. 2; Additional file 1: Table S2). The *EgrG_09018* gene probably gave rise to *EgrG_10769* and *EgrG_7258* (and by extension, their orthologs) by retrotransposition-mediated duplication. The absence of orthology between Cestoda and Trematoda retrocopies of *EgrG_09018* (and its orthologs) means that they likely represent a recent duplication event, which occurred after the split of the lineages of these classes. In the same way, many USP copies emerged independently from *EgrG_09018*, *EgrG_06206*, and *EgrG_20248*, resulting in class-specific clades for the Trematoda and Cestoda (Fig. 2). Most of the USP genes that form local clusters in the genome, are also grouped together in the phylogenetic tree. This applies to *EgrG_08734*, *EgrG_08736*; *EgrG_08738*, and their orthologs in the Cestoda; *Csin107893*, *Csin107894*, *Csin107895,* and their orthologs in *O. viverrini*; and *Smp_13687* and *Smp_13689* and their orthologs in *S. haematobium* (Figs. 1a and 2). Interestingly, *EgrG_08738* is phylogenetically close to *EgrG_20190* (named *EgrG_ps1* in Fig. 1b). The latter one may therefore be a pseudogene that emerged from *EgrG_08738*. Along the same line, all other USP paralogs located in the same cluster (Figs. 1 and 2) could have arisen from *EgrG_08738* or *EgrG_20190* by successive tandem duplications. This observation could be extended to the other Cestoda species. Based on the organization of USP genes within the genome and on their phylogenetic relationships, an array of tandem and retrotransposition duplication events might be inferred for the Trematoda and Cestoda classes. This latter mechanism seems to have played a pivotal role in the emergence of USP genes in the free-living flatworm *S. mediterranea*, whose USP gene tree exhibits a star-like topology with few ancestral genes and many locally isolated USP paralogs distributed throughout the genome.

### DNA motif analysis

DNA motif analysis of the promoter regions detected the heptanucleotide -*CCAATCA*- between positions − 200 and − 40 upstream for almost all USP genes (Additional file 1: Table S3). This motif is a known DNA binding site for the mammalian nuclear transcription factor Y (NF-Y, HAP in *S. cerevisiae*), which promotes the initiation of gene transcription [52]. NF-Y consists of three subunits: NFA, NFB and NFC (HapB, HapC, and HapE orthologs in *S. cerevisiae*). In the presence of reactive oxygen species (ROS), oxidized HapC prevent the interaction with the HapE and HapB subunits. Consequently, the formation of the CCAAT-binding complex is abolished and their nuclear localization and regulation of target genes becomes affected [53]. Using the Pfam numbers PF02045 and PF00808, we identified the orthologs of NFYA, NFYB, and NFYC for the species studied (data available at the WormBase database). In line with this, the GOMo tool reports that the motif -*CCAATCA*- is involved in the oxidation-reduction processes as the ATP synthesis coupled to proton transport. In both Cestoda and Trematoda classes, some genes lack the conserved -*CCAATCA*−/-*CCAAT*- motif (e.g. *EgrG_02019*, *EgrG_08735*, *EgrG_09839*, and their orthologs). This could suggest a common origin for these genes, different regulation properties, or evidence of a pseudogenization process. In other genes (e.g. *EgrG_08736*, *EgrG_08738*, *EgrG_07258, EgrG_10769, EgrG_09018,* and orthologs), the -*CCAATCA*- motif occurs at the exact same position. These data provide insights about the functional diversification of the USP promoter regions and their origins by retrotransposition or tandem duplication events, as well as about the relationship between these last two.

### 3D protein modeling

The highest scoring template in the 3D structural analysis of EgrG_08736 was the USP MJ0577 from *Methanococcus jannaschii*, with a confidence of homology of 99.9%. Against this template, the alignment coverage was 84%, and the sequence identity between both proteins was 33%. Analysis with ModFOLD v4.0 returned a global quality model score of 0.7 and a *p*-value of 6.4E-4 (Additional file 3: Figure S3A). The presence of a large coil between the second beta strand and the second alpha helix (Additional file 3: Figure S3B) is due to the insertion of eleven amino acids in our query sequence relative to the template. This region is highly variable across USP paralogs [5] (Additional file 3: Figure S4); it is located on the outside of the protein pocket in the 3D model (Additional file 3: Figure S3). Using Phyre v2 Investigator, we predicted the likely functional sites in our model, as well as the effect of mutations at these specific sites (described below; see also Additional file 3: Figure S3C).

### Gene expression analysis

**Fig. 3:**
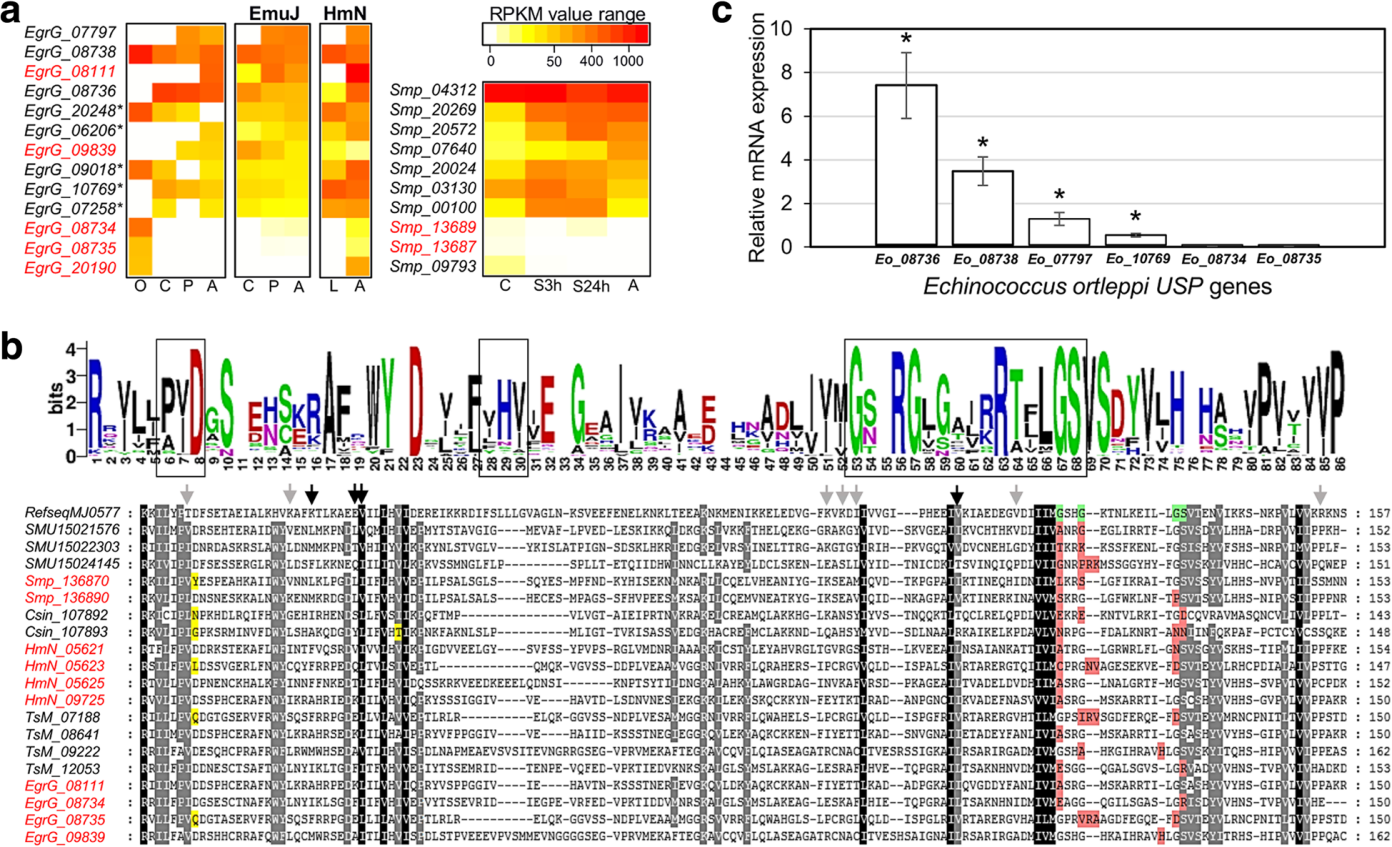
Relationship between USP sequence modifications and gene expression patterns. **a** The gene expression profile is highly variable between the different life cycle stages of Cestoda and Trematoda species, with some USP genes with null or very low expression (*EgrG_08734*, *EgrG_08735* and orthologs), others expressed in specific manner (*EgrG_08111; EgrG_07797* and orthologs), and others constitutively expressed (*EgrG_08738* and orthologs). ID numbers in red color refer to USP proteins that exhibit modifications in the ATP binding motif. The asterisk refers to the “ancestral” USPs (see Fig. 2). **b** USP sequence variations in the Platyhelminthes. On top, sequence logo generated with all USP sequences without changes to the ATP binding motif [Gx2Gx9G(S/T)]. Amino acids interacting with ligands are shown in boxes. Below, alignment of USP protein sequences showing modifications in the protein motif. ID numbers (in red) refer to USP proteins for which RNA-seq data was available, to facilitate the comparison between sequence modification and gene expression patterns. Modifications in the [Gx2Gx9G(S/T)] motif and at other sites known to be involved in ligand interaction are highlighted in red and yellow, respectively. The sequence of the *Methanococcus jannaschii* USP MJ0577 USP was used as a reference (starting from position 6), with the ATP binding motif highlighted in green. The residues under functional divergence are indicated by arrows (black arrows, residues shared by Cestoda and Trematoda; gray arrows: residues specific to the Cestoda or Trematoda) (see Table 1). **c** qPCR gene expression analysis of USP genes in the pre-adult form of *E. ortleppi.* Some genes (*E0_08736*, *Eo_08738*, *Eo_07797*, and *Eo_10769*) showed higher levels of gene expression than others (*Eo_08734* and *Eo_08735*), in line with previously published RNA-seq data for the genus *Echinococcus* (see above). Asterisks indicate a *p*-value < 0.01 for the comparison of *Eo_08734* and *Eo_08735* with the other genes

We evaluated RNA-seq data from previous transcriptomic reports [21, 22, 50]. Although these data do not include all developmental stages for each species, they are representative of the entire parasitic life cycle, occurring in both the intermediate and the definitive host. USP gene expression is highly variable between different life cycle stages, and while some genes are expressed constitutively (*Smp_04312*; *EgrG_08738* and their orthologs in *E. multilocularis* and *H. microstoma*), others are expressed in a specific spatio-temporal manner (*Smp_07640* and *Smp_09793*; *EgrG_08734*, *EgrG_08111*, and their orthologs in *E. multilocularis* and *H. microstoma*) (Fig. 3a). As described above, *EgrG_02019* contains an *indel* that generates a stop codon in *Echinococcus* spp. (Fig. 1b). Because this gene also exhibits a null or very low expression in all life cycle stages, we consider it a pseudogene with residual transcriptional activity. Interestingly, the gene expression patterns of *EgrG_08734* and *EgrG_08735* are almost identical to those of *EgrG_02019* and its orthologs in the Cestoda, as they are expressed only in the oncosphere stage, and only at very low levels (Fig. 3a). In addition, the translation product of *EgrG_02019* (after including the thymine at position 61 in the coding sequence, see Fig. 1b) has an amino acid sequence variation at the ATP-binding motif (GS > DN; not shown). In the same manner, EgrG_08734 and EgrG_08735 show modifications at these sites, GS > GR for the former, and GS > DS for the latter (Fig. 3b). Our protein modeling predicts that these changes are critical for ligand binding and may have a negative impact on protein function (Additional file 3: Figure S3C). Moreover, Smp_13687 and Smp_13689 are the only two USPs in *S. mansoni* showing mutations within the ATP-binding motif, which also had a very low or null gene expression in all life cycle stages (cercaria, schistosomula, adult; see Fig. 3a and b). Although there are no gene expression data for the other species (e.g. *E. canadensis*, *C. sinensis*, *T. solium*, etc.), they shared several amino acid substitutions at sites predicted to bind the ATP molecule (Fig. 3b). To validate the RNA-seq data for the genus *Echinococcus*, we performed real time PCR of six USP genes from the pre-adult form of *Echinococcus ortleppi*. As expected, *Eo_08734* and *Eo_08735* were expressed at a very low level (< 1/10 of *Eo_08736* expression and barely detectable in the qPCR curves; see Fig. 3c). On the other hand, *Eo_10769*, *Eo_07797* and *Eo_08738* were expressed at medium levels, and *Eo_08736* was highly expressed (Fig. 3c). These results indicate a positive correlation between amino acid substitutions at sites that are critical for the contact with ligands and gene expression levels in the different life cycle stages of Platyhelminthes species.

### Positive selection and divergence analysis

Using 63 coding sequences from species of the class Cestoda (codon alignment in Additional file 3: Figure S5), we detect several sites under positive selection with PAML (Table 1). At first, these residues do not correspond to specific sites for the interaction with ligands, however, they are located in the vicinity (e.g. sites 7 K, 16E, 20 T, 32 K, 114 K, 115I, 117E, 120G, and 151 N) (Table 1). In four amino acids (20 T, 32 K, 80E, and 151 N), the posterior probability were more than 99%, suggesting an important role as adaptive sites in the evolution of the USP genes. Using Selecton 7, which ranks the residues from 1 to 7 (values 1 and 2 means Ka/Ks ratio > 1, and values from 3 to 7 denotes Ka/Ks ratio > 1), there was no signal of positive selected sites. However, we detected several residues with the value of 3 (Ka/Ks ratio near 1) (Additional file 1: Table S4). Most of these sites correspond to the positive selected sites found with PAML (Table 1, and Additional file 1: Table S4). These results suggest the presence of highly divergent sequences with adaptive sites in the USP genes of species of class Cestoda.

**Table 1:** Positive selection analysis of USP genes for species of class Cestoda

Model	Estimates of parameters	*L*	Positive selected sites (PSS)^a^
M0 (one-ratio)	*ω* = 0.32480	−17568.832757	None
M1a (neutral)	*ω*_0_ = 0.03822, *ω*_1_ = 1, *p*_0_ = 0.95404, *p*_1_ = 0.04596	−16175.637618	Not allowed
M2a (selection)	*ω*_0_ = 0.03823, *ω*_1_ = 1., *ω*_2_ = 1, *p*_0_ = 0.95404, *p*_1_ = 0.00224, *p*_2_ = 0.04372	−16175.637618	151 N ^b^
M7 (beta)	*p* = 0.04543, *q* = 0.40703	−16106.206229	Not allowed
M8 (beta & *ω*)	*p*_0_ = 0.97089, *p* = 0.03908, *q* = 0.98312, *p*_1_ = 0.02911, *ω* = 1	−16028.140026	7 K 16E **20 T 32 K** 45R 48 K 49 K 50R 51D 64 K 65S 671 N 72E 77 L **80E** 83 N 114 K 115I 117E 120G **151N**

^a^Positive selected sites (Bayes Empirical Bayes, BEB) are inferred at a cutoff posterior probability *P* ≥ 95%. Values for *P* ≥ 99% are shown in bold font. The underlined PSS indicate a value of 3 (range from 1, positive selection, to 7, purifying selection) obtained with SELECTON (see Additional file 1: Table S4). Amino acid sites correspond to the reference sequence MJ0577 from *Methanococcus jannaschii*

^b^Despite the presence of positive selected sites (24 sites with *P* ≥ 50%, 1 site with *P* ≥ 95%), the LRT test was not significant when comparing the Log likelihood scores from the M1a and M2a models

At the same time, based on the protein sequences, we searched for functional divergence between paralogs in the Cestoda and Trematoda classes using Diverge v3.0. The results are summarized in Table 2. In general, we found large differences within both Cestoda and Trematoda clusters, from one or few sites to numerous sites under functional divergence (Table 2). Some clusters where genes are located in tandem (Ce8_08736 and Ce5_G08738; Tr3_CsinT265 and Tr4_CsinT265) have no or just one site showing functional divergence; however, we found the opposite for other cluster comparisons (Ce4_08735 and Ce8_08736; Tr4_CsinT265 and Tr5_CsinT265) (Table 2). Functionally divergent amino acids are not restricted to a specific site, but are instead spread over a number of sites near the ATP-binding motif (see Table 2 and Fig. 3b for the reference sequence). However, several amino acid positions (e.g. 29, 35, 113) were more frequent than others. A minor number of clusters comparisons (6/66 for Cestoda, and 14/55 for Trematoda) did not show functional divergence. These results indicate the presence of distinct levels of functional divergence between several USP clusters within Cestoda and Trematoda classes.

**Table 2:** Functional divergence analysis (Type I) within Cestoda and Trematoda species

Cestoda comparisons^a^				
Cluster 1	Cluster 2	*Ɵ* ± SE	LRT	Sites (Q_k_ > 0.9)^c^
Ce11_07258	Ce12_09018	0.70 ± 0.14	23.04	28,31,37,44,94,98,124
Ce10_10769	Ce12_09018	0.78 ± 0.17	20.59	14,**29**,96,98,115,120,159
Ce11_07258	Ce10_10769	0.95 ± 0.16	31.63	Almost all
Ce4_08735	Ce3_09839	0.97 ± 0.14	45.46	Almost all
Ce4_08735	Ce5_08738	0.98 ± 0.17	32.99	Almost all
Ce4_08735	Ce6_08734	0.91 ± 0.14	40.82	Almost all
Ce4_08735	Ce8_08736	1.36 ± 0.15	80.72	Almost all
Ce5_08738	Ce6_08734	0.80 ± 0.18	18.96	7,**35**,**36**,39,45,59,98,100,108,**113**,121
Ce5_08738	Ce8_08736	0.64 ± 0.14	18.81	**35**
Ce6_08734	Ce8_08736	0.79 ± 0.14	29.00	19,**36**,72,100,**113**,121,159
Trematoda comparisons^b^				
Cluster 1	Cluster 2	*Ɵ* ± SE	LRT	Sites (Q_k_ > 0.9)^c^
Tr4_CsinT265	Tr3_CsinT265	0.64 ± 0.20	9.84	None
Tr3_CsinT265	Tr5_CsinT265	0.75 ± 0.21	12.58	134
Tr4_CsinT265	Tr5_CsinT265	0.99 ± 0.24	16.21	Almost all
Tr2_SmpA	Tr3_CsinT265	0.94 ± 0.13	45.93	Almost all
Tr2_SmpA	Tr5_CsinT265	0.68 ± 0.17	15.36	**29**
Tr2_SmpA	Tr1_SmpA	0.69 ± 0.18	14.17	12,26,102
Tr7_CsinT265SmpA	Tr1_SmpA	0.68 ± 0.19	12.56	12,26,102
Tr6_CsinT265SmpA	Tr9_CsinT265SmpA	0.84 ± 0.19	17.70	12,**29**,32,**35**,**36**,59,110,**113**,114,117,118,122,144,145,150

^a^Cestoda clusters are defined based on the *E. granulosus* IDs, e.g. the cluster Ce3_09839 is composed by the EgrG_09839, EmuJ_09839, Ecan_08199, TsM_09222, and HmN_01226 sequences

^b^Trematoda clusters are described as follows: Tr1 (Smp_136870, A_04288, Smp_136890, A_06342), Tr2 (Smp_043120, A_03767, Smp_202690, A_04393), Tr3 (Csin107893, T265_02180, CsinSc585new, T265_02179), Tr4 (Csin107892, Csin110039, Csin107891, T265_02178a, T265_02178b,T265_02177), Tr5 (Csin107894, T265_02181, Csin107895, T265_02182), Tr6 (Smp_076400, A_07834, Csin112002, T265_05585), Tr7 (Smp_031300, A_04567, Csin112617, T265_03499), Tr9 (Smp_001000, A_07787, Smp_200240, A_05680)

^c^Amino acid sites correspond to the reference sequence MJ0577 from *Methanococcus jannaschii*. Amino acids shared by Cestoda and Trematoda species are indicated in bold font and plotted in the Fig. 3b. Underlined sites correspond to positive selected sites detected with PAML (Table 1)

## Discussion

The expansion of gene families by gene duplication represents a successful strategy for the propagation of gene copies through the acquisition of specialized or novel functions (e.g. globin or homeobox gene families) [54, 55]. Although some genes may acquire adaptive novelties that are maintained from one generation to the next, others may follow a pseudogenization process through the accumulation of deleterious mutations. An understanding of when and how fast these duplications occur is key to our understanding of the duplicated genes’ functional diversity.

Here, we explored the evolutionary fates of the USP gene family in Platyhelminthes species of medical relevance. We found that the USP genes of this phylum are mostly intronless, transcribed independently and encoding a single protein domain. A few USPs (*EgrG_09018* and orthologs) contain a single intron in a conserved position around amino acid 75. This is similar to what has been described for *Hydra*, where 22 out of 24 USP genes are intronless [5]. Based on a well-supported monophyletic clade, Forêt and colleagues consider that a single retrotransposition event had a pivotal role in the emergence of most intronless USP genes after the anthozoan/hydrozoan divergence [5]. Our results show that the same process could have been very important after the separation of the Cestoda and Trematoda classes. Nevertheless, the cluster organization of the USP genes in the Platyhelminthes (around 50% of USP genes occur in clusters in both Cestoda and Trematoda) revealed the importance of tandem duplications for the generation of new USP copies. This idea is supported by the presence of a well-conserved DNA motif occurring at the same position in tandemly organized genes (e.g. *EgrG_08736*, *EgrG_08738,* and orthologs in the Cestoda; *T265_02179* and *T265_02181*, *Smp_001000* and *Smp_200240*, and orthologs in *C. sinensis* and *S. haematobium*, respectively). Surprisingly, we found that the position of the -*CCAATCA*- motif was also preserved between isolated USP genes and their most closely related homologs (e.g. *EgrG_10769* and *EgrG_07258*, which probably emerged from *EgrG_09018*), suggesting a retrotransposition event that included both the coding sequence and promoter region. This might be due to the fact that the transcription start sites (TSS) tend to be interspersed rather than located at one specific site. If a TSS upstream of the promoter region is used, a large part of the core promoter may be transcribed [56, 57]. This mechanism could ensure the transcriptional activity of the newly retrotransposed genes and would consequently avoid the effects of neutral evolution, i.e., the accumulation of deleterious mutations.

USPs are often classified based on the presence or absence of the conserved ATP-binding motif residues [58]. A positive correlation between the conservation of the [Gx2Gx9G(S/T)] protein motif and crystal solubility in the presence of ATP, or an ATP analog, has been previously described [11]. In addition, the same authors found a high level of conservation (~ 80%) at amino acid positions forming part of the motif across all crystals extracted from the PDB database, with exception of the second glycine (G130, which is preserved in 50% of crystals). Our protein model showed that amino acid alterations at specific ligand-binding sites have a negative effect on protein function, including modifications at P11, D13, V41, G127, G130, G140, and S/T141. The residue G130 was the most exchangeable amino acid, and could thus be most easily substituted non-synonymously (Additional file 3: Figure S3C). The USP gene expression data allowed us to associate the transcriptional activity with modifications at residues that are critical for ligand interaction. In general, we observed that alterations in the [Gx2Gx9G(S/T)] motif and at other positions within the protein pocket (e,g, D13, V41) are associated with very low or null levels of gene expression in almost all life cycle stages of the parasites, probably as a result of functional redundancy [59]. Additionally, for several USPs, we observed amino acid insertions (*SMU15024145*, *EgrG_08735*, *HmN_05623*, etc.) and deletions (*T265_02176*, *HmN_00323*, *Csin110041*) within the [Gx2Gx9G(S/T)] motif. These modifications could lead to a steric hindrance, thereby preventing the contact with the ligand. Based on this, we believe that several USP genes in the Cestoda (*EgrG_08734*, *EgrG_08735*, and orthologs; *HmN_05021* and *HmN_05022*; etc.) and Trematoda (*Smp_136870*, *Smp_136890*; *Smp_09793* and their orthologue *A_04226*; etc.) are in the process of pseudogenization. Like the pseudogenes described here (Fig. 1a and b), the genes under pseudogenization lack the canonical and well conserved -*CCAATCA*- motif in the promoter region, which probably affects their transcriptional activity. Although less likely, the possibility that changes in the ATP-binding sites might expand the ligand repertoire should not be dismissed. Further functional studies will be necessary to clarify these findings.

**Fig. 4:**
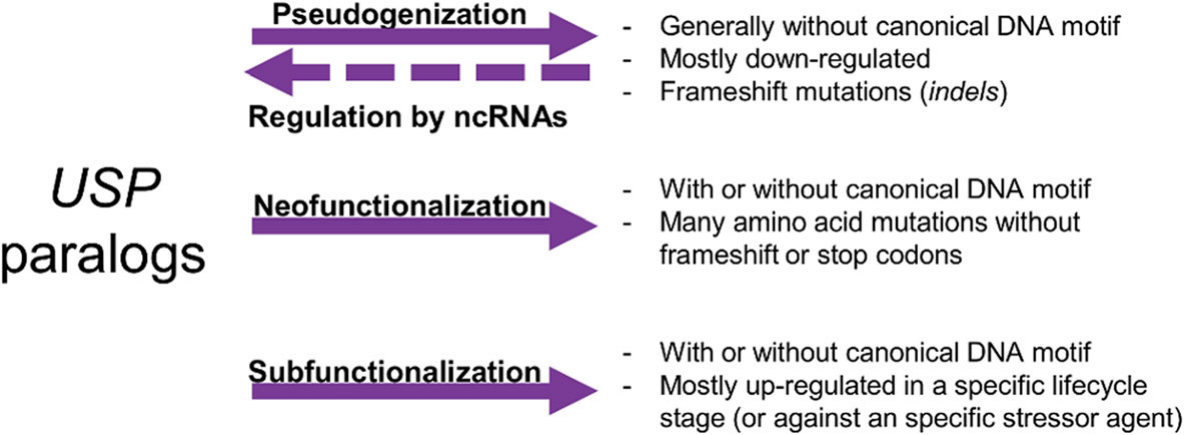
Possible evolutionary fates for the USP paralogs in Platyhelminthes parasites. First, a new *USP* copy can accumulate deleterious mutations, leading to alterations in the protein sequence with a loss of function (pseudogenization). From this, some ncRNAs can be transcribed, and thus, regulate the gene expression of the other USP paralogs via mRNA degradation (regulation by ncRNAs). Second, the USP paralog could undergo several non-synonymous mutations, thereby acquiring a new function (neofunctionalization). Finally, some *USP* copies could maintain the same function, but be expressed in a specific life cycle stage or in response to a specific stressor (subfunctionalization).

Despite several gene losses in species of the *Taeniidae* family, we found a high functional divergence between the different USP paralogs. Positively selected sites identified by PAML (Table 1) included several residues near to the ligand binding sites, a similar result observed by Diverge v3.0 (Table 2). Several amino acids (e.g. 7 K, 32 K, 114 K, 117E), were identified by the two approaches, suggesting an important role for these sites in the evolution of the USP genes. The number of sites under functional divergence was highly variable regarding cluster comparisons (Table 2). This dynamic behavior could indicate that some USP genes are evolving faster than others. Interestingly, no amino acid changes were observed in the ATP-binding motif of the USP genes shared by all species studied (“ancestral” USPs, see Fig. 3). Moreover, the lack of the *-CCAATCA-* motif in promoter regions is restricted to the non-ancestral USPs. These observations may suggest the maintenance of ancient functions for the “ancestral” USPs, and the emergence of functional novelties (through recurrent mutations) for the “new” USP paralogs. Functional divergence is observed in a greater number of residues, but some amino acid positions (29, 35, 36, 113) are shared by several cluster comparisons. The repeated occurrence of these sites may represent an adaptive trait for the protein function. The high divergence agrees with the multifunctional behavior observed for several taxa. In *E. coli*, for example, the *uspC* and *uspE* both affect motility positively and adhesion negatively, while *uspA* and *uspD* are involved in the oxidative stress defense [7]. In addition to changes in the protein coding sequence, selective forces can lead to changes in the regulatory elements. For example, in *Arabidopsis thaliana*, a high divergence between duplicated copies was observed for the *cis*-regulatory elements and methylation patterns, leading to different expression profiles [60]. These findings suggest that different USP genes may be subjected to dissimilar types of selective pressure. Some genes will accumulate deleterious mutations both in the promoter regions (affecting their transcriptional activity) and in the protein coding sequence (generating a truncated and/or non-functional product). These genes will be under neutral evolution and become pseudogenized as a consequence of functional redundancy (Fig. 4). Even so, there might be a possible role for the residual transcripts of genes under pseudogenization, including *EgrG_USPps1*, its orthologs in *E. multilocularis* and *E. canadensis,* and other genes (see above): in a novel mechanism of gene regulation by pseudogenes, these non-coding RNA (ncRNA) transcripts might act as gene expression regulators by promoting the degradation (or kidnapping) of functional USP mRNAs by hybridization (Fig. 4) [61]. This mechanism might be of relevance in the context of the narrow spatiotemporal regulation of the USP genes in each life cycle stage.

In contrast to the process of pseudogenization, many USP paralogs may have acquired new functions, leading to functional diversification within the gene family. Since several amino acid modifications have occurred close to ligand-binding sites, this functional diversification may be associated to the interaction with different types of ligands. Furthermore, several USPs were found to occur as dimers or higher oligomeric complexes [8, 11], suggesting that substitutions involved in the protein oligomerization could increase the complexity of the protein-protein interactions. These sequence variations, and those found in promoter regions, could be considered adaptive traits that emerge as part of subfunctionalization or neofunctionalization processes (Fig. 4). The publication of the genome sequences of several Platyhelminthes species [21, 22, 50, 62] revealed that gene expansion, such as in heat shock proteins, species-specific antigens, or proteases, is a widespread process related to the adaptation to parasitism. In this way, some of the USP paralogs could be considered adaptations to the parasitic lifestyle by increasing the repertoire of binding proteins, by establishing complex protein-protein interactions (homo- and heterodimers), or by being expressed in a specific tissue, life cycle stage, or in response to a particular stressor.

## Conclusions

In the present work we found that the USP gene family has an ancient origin and follows a complex evolutionary pattern (pseudogenization and sub/neofunctionalization) for several Platyhelminth species. This scenario may result from different selective pressures acting on the USP genes. If these patterns are restricted to parasitic flatworms, or also include the free-living species, remains to be elucidated. Further studies associating functional diversity with the various sequence modifications will help deepen our knowledge about the patterns and regulation of USP gene expression. Additional analyses will be necessary to investigate the role of ncRNAs in the specific spatiotemporal regulation of the USP genes.

## Additional files

Additional file 1: Table S1. Details of each primer designed for the six USP genes. **Table S2.** USP orthologues relationship between Platyhelminthes species. **Table S3.** Motif scanning in promoter regions of the USP genes. **Table S4** SELECTON results for Cestoda species. **Table S5.** IDs used for *M. lignano* and *G. salaris* in the phylogenetic analysis. (XLSX 37 kb)

Additional file 2: Protein and upstream sequences. (TXT 117 kb)

Additional file 3: Figure S1. Phylogenetic tree generated using the aLRT-SH method with PhyML. **Figure S2.** Phylogenetic tree generated using Bayesian Inference with BEAST v1.8.4. **Figure S3.** EgrG_08736 3D protein modeling. **Figure S4.** Protein alignment used to generate the phylogenetic trees. **Figure S5.** Positive selection analysis in Cestoda species. (PPTX 8391 kb)
