# Supplementary material for: Evolutionary fates of universal stress protein paralogs in Platyhelminthes
Source: BMC Evol Biol. 2018 Feb 1;18:10. doi: 10.1186/s12862-018-1129-x (PMC5793430; doi:10.1186/s12862-018-1129-x)
Supplement: Supplementary file 3 — Phylogenetic tree generated using the aLRT-SH method with PhyML. Figure S2. Phylogenetic tree generated using Bayesian Inference with BEAST v1.8.4. Figure S3. EgrG_08736 3D protein modeling. Figure S4. Protein alignment used to generate the phylogenetic trees. Figure S5. Positive selection analysis in Cestoda species. (PPTX 8391 kb) [file 12862_2018_1129_MOESM3_ESM.pptx]

## Slide 1
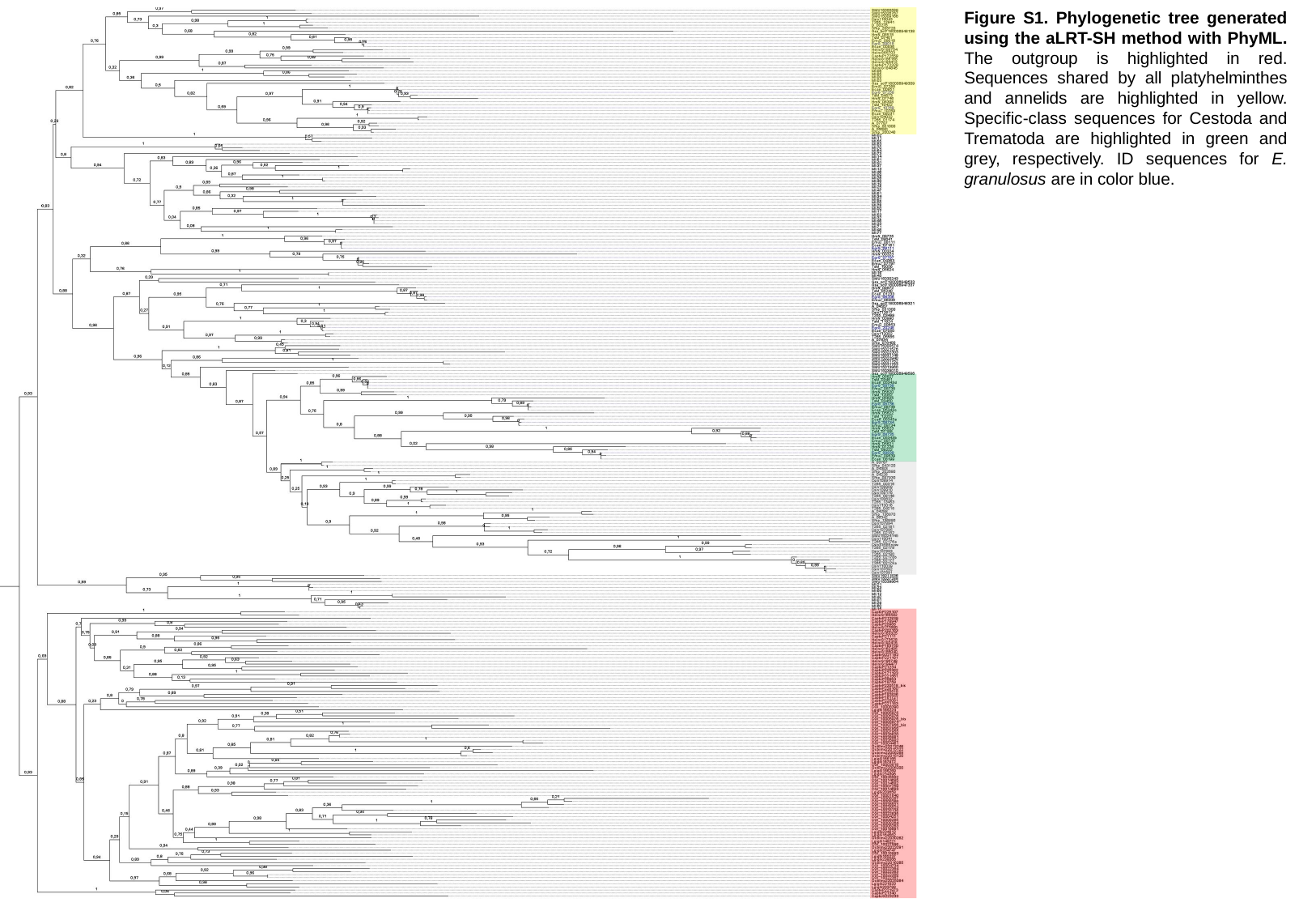

Figure S1. Phylogenetic tree generated using the aLRT-SH method with PhyML. The outgroup is highlighted in red. Sequences shared by all platyhelminthes and annelids are highlighted in yellow. Specific-class sequences for Cestoda and Trematoda are highlighted in green and grey, respectively. ID sequences for E. granulosus are in color blue.

## Slide 2
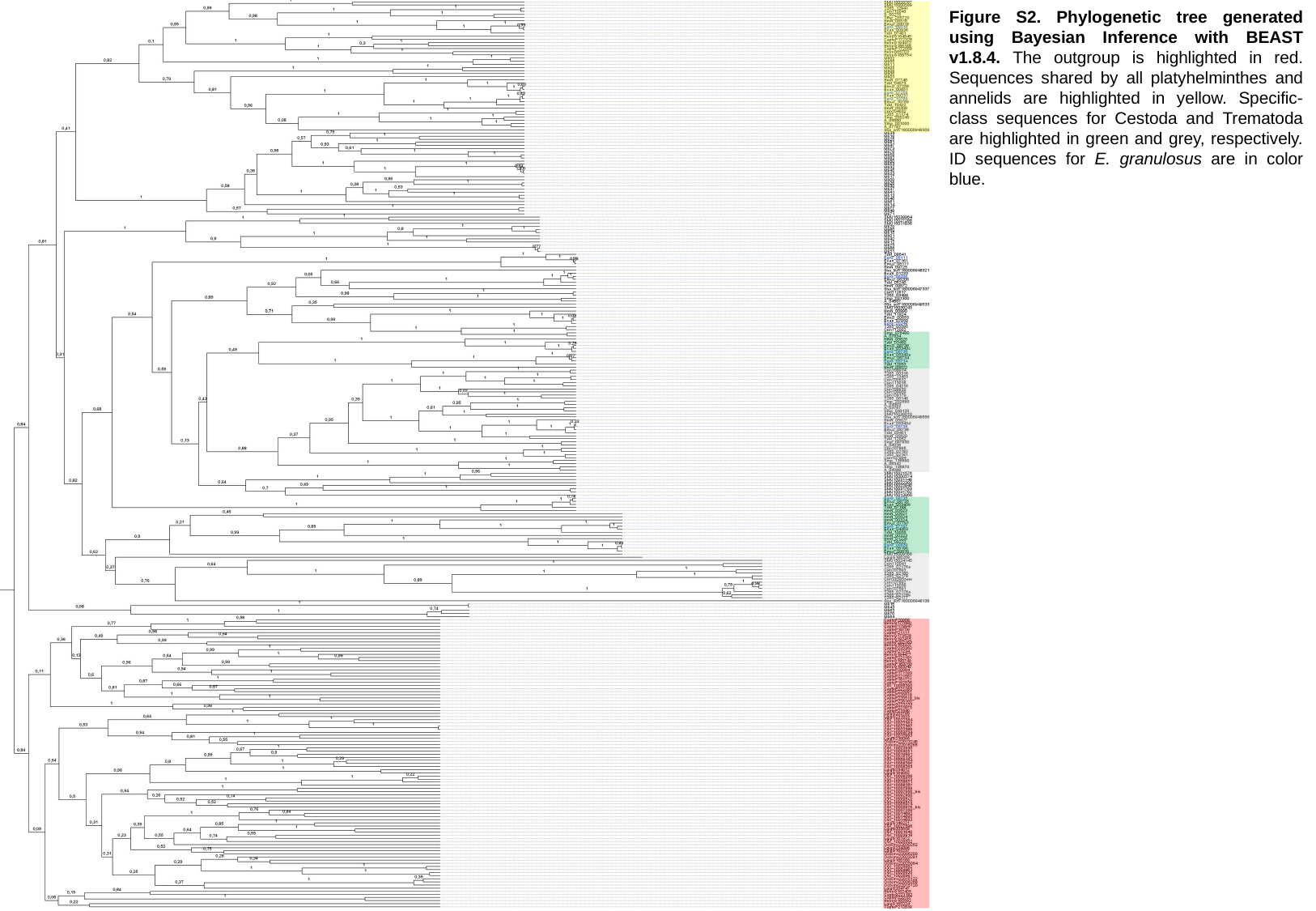

Figure S2. Phylogenetic tree generated using Bayesian Inference with BEAST v1.8.4. The outgroup is highlighted in red. Sequences shared by all platyhelminthes and annelids are highlighted in yellow. Specific-class sequences for Cestoda and Trematoda are highlighted in green and grey, respectively. ID sequences for E. granulosus are in color blue.

## Slide 3
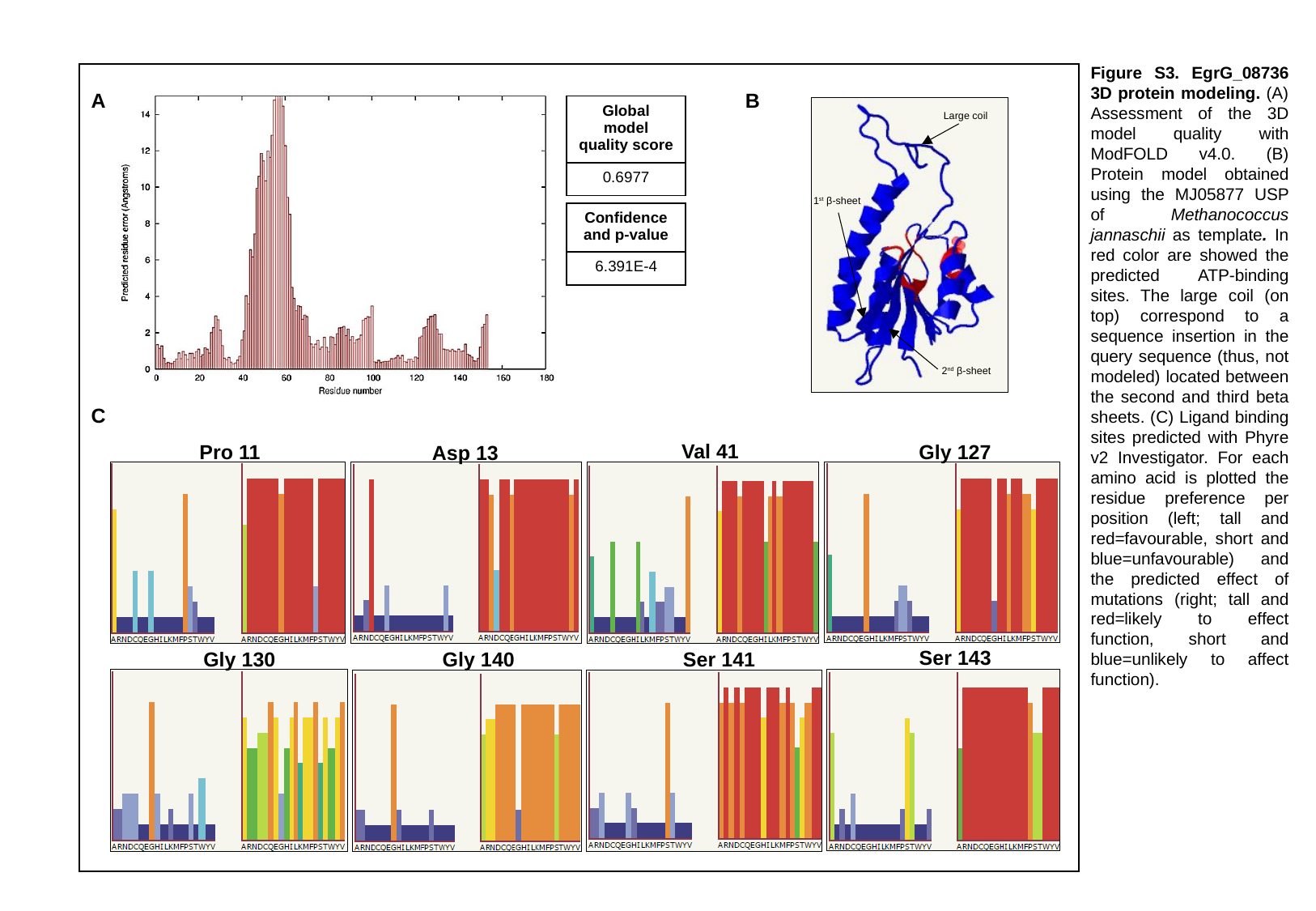

Figure S3. EgrG_08736 3D protein modeling. (A) Assessment of the 3D model quality with ModFOLD v4.0. (B) Protein model obtained using the MJ05877 USP of Methanococcus jannaschii as template. In red color are showed the predicted ATP-binding sites. The large coil (on top) correspond to a sequence insertion in the query sequence (thus, not modeled) located between the second and third beta sheets. (C) Ligand binding sites predicted with Phyre v2 Investigator. For each amino acid is plotted the residue preference per position (left; tall and red=favourable, short and blue=unfavourable) and the predicted effect of mutations (right; tall and red=likely to effect function, short and blue=unlikely to affect function).
B
A
| Global model quality score |
| --- |
| 0.6977 |
Large coil
1st β-sheet
2nd β-sheet
| Confidence and p-value |
| --- |
| 6.391E-4 |
C
Val 41
Pro 11
Gly 127
Asp 13
Ser 143
Gly 130
Gly 140
Ser 141

## Slide 4
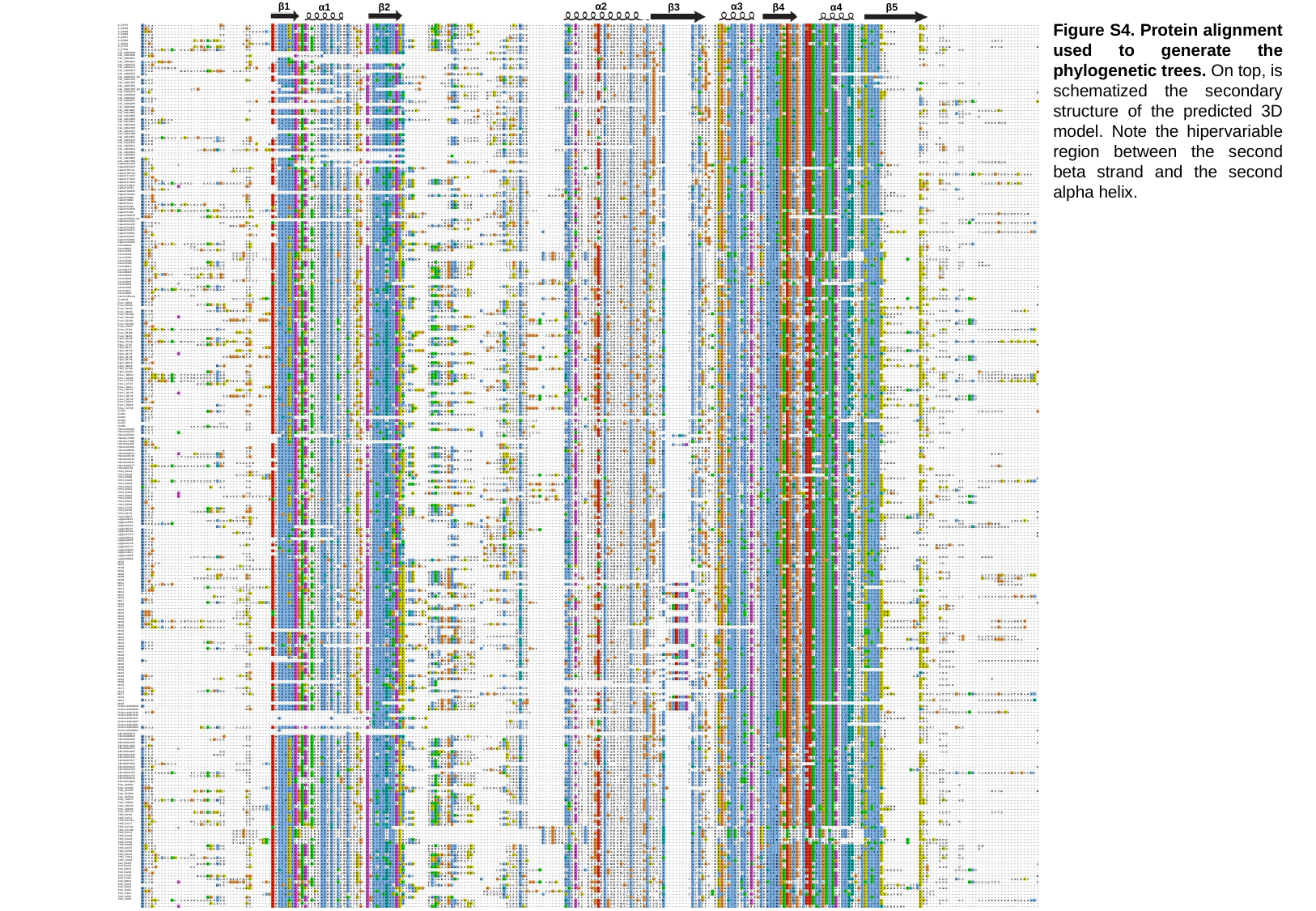

Figure S4. Protein alignment used to generate the phylogenetic trees. On top, is schematized the secondary structure of the predicted 3D model. Note the hipervariable region between the second beta strand and the second alpha helix.
α3
α2
β1
β2
α1
α4
β3
β4
β5
A_03767
A_04226
A_04288
A_04393
A_04567
A_05680
A_06342
A_07787
A_07834
CGI_10001640
CGI_10003818
CGI_10004251
CGI_10004461
CGI_10004734
CGI_10005974
CGI_10005975
CGI_10005976
CGI_10005976_bis
CGI_10007259
CGI_10007955
CGI_10007956
CGI_10007956_bis
CGI_10008263
CGI_10008264
CGI_10008265
CGI_10008267
CGI_10008268
CGI_10008390
CGI_10014682
CGI_10014683
CGI_10014685
CGI_10015993
CGI_10019991
CGI_10022383
CGI_10022384
CGI_10022387
CGI_10022388
CGI_10022635
CGI_10025135
CGI_10026502
CGI_10026521
CGI_10026523
CGI_10026620
CGI_10026981
CGI_10026982
CGI_10027596
CapteG221182
CapteG223233
CapteP161721
CapteP163750
CapteP171183
CapteP172328
CapteP172559
CapteP178947
CapteP18759
CapteP192836
CapteP202163
CapteP20968
CapteP20984
CapteP21111
CapteP21234
CapteP212839
CapteP21340
CapteP220518
CapteP220518_bis
CapteP221181
CapteP221183
CapteP221561
CapteP221973
CapteP225081
CapteP225107
CapteP225362
CapteP228160
Csin100632
Csin104032
Csin107891
Csin107892
Csin107893
Csin107894
Csin107895
Csin108914
Csin109179
Csin109909
Csin109910
Csin110039
Csin110041
Csin110043
Csin112002
Csin112617
Csin113016
CsinSc585new
D_00276
Ecan_00851
Ecan_00936
Ecan_01233
Ecan_04363
Ecan_05343a
Ecan_05343b
Ecan_05343c
Ecan_05343d
Ecan_07050
Ecan_07161
Ecan_08199
Ecan_09231
EgrG_06206
EgrG_07258
EgrG_07797
EgrG_08111
EgrG_08734
EgrG_08735
EgrG_08736
EgrG_08738
EgrG_09018
EgrG_09839
EgrG_10769
EgrG_20248
EmuJ_00653
EmuJ_06206
EmuJ_07258
EmuJ_07797
EmuJ_08111
EmuJ_08734
EmuJ_08735
EmuJ_08736
EmuJ_08738
EmuJ_09018
EmuJ_09839
EmuJ_10769
Gsa01
Gsa02
Gsa03
Gsa04
Gsa05
Gsa06
HelroG162405
HelroG163325
HelroG165592
HelroG173528
HelroG177680
HelroG184845
HelroG185040
HelroG186168
HelroG188754
HelroG190749
HelroG192415
HelroG194412
HelroG194477
HelroG65703
HmN_00323
HmN_00324
HmN_00990
HmN_01226
HmN_05620
HmN_05621
HmN_05622
HmN_05623
HmN_05624
HmN_05625
HmN_05627
HmN_06308
HmN_07746
HmN_08518
HmN_09725
HmN_09872
LgigG149221
LgigG164562
LgigG165224
LgigG165237
LgigG166166
LgigG167972
LgigG186269
LgigG203658
LgigG203798
LgigG204747
LgigG231833
LgigG234012
LgigG234396
LgigG238066
Mli01
Mli02
Mli03
Mli05
Mli06
Mli08
Mli09
Mli11
Mli12
Mli13
Mli14
Mli15
Mli16
Mli17
Mli18
Mli21
Mli23
Mli25
Mli26
Mli29
Mli30
Mli32
Mli33
Mli34
Mli37
Mli41
Mli42
Mli43
Mli45
Mli46
Mli47
Mli48
Mli50
Mli53
Mli55
Mli58
Mli60
Mli61
Mli63
Mli64
Mli68
Mli70
Mli71
Mli73
Mli77
Mli78
Mli81
Mli82
Ocbimv22006200
Ocbimv22006268
Ocbimv22013248
Ocbimv22015120
Ocbimv22015122
Ocbimv22016265
Ocbimv22023291
Ocbimv22025064
Ocbimv22030262
SMU15000574
SMU15000589
SMU15009166
SMU15011836
SMU15013960
SMU15021576
SMU15022303
SMU15022940
SMU15024145
SMU15026767
SMU15027288
SMU15030243
SMU15031148
SMU15031762
SMU15031763
SMU15039010
SMU15039964
Smp_001000
Smp_031300
Smp_043120
Smp_076400
Smp_097930
Smp_136870
Smp_136890
Smp_200240
Smp_202690
Smp_205720
T265_00316
T265_01174
T265_02176a
T265_02177
T265_02178a
T265_02178b
T265_02179
T265_02180
T265_02181
T265_02182
T265_03499
T265_04215
T265_05585
T265_08146
T265_12941
T265_13453
TsM_03450
TsM_03451
TsM_04573
TsM_05240
TsM_07188
TsM_07401
TsM_08641
TsM_09222
TsM_10056
TsM_10422
TsM_11924
TsM_12052
TsM_12053

## Slide 5
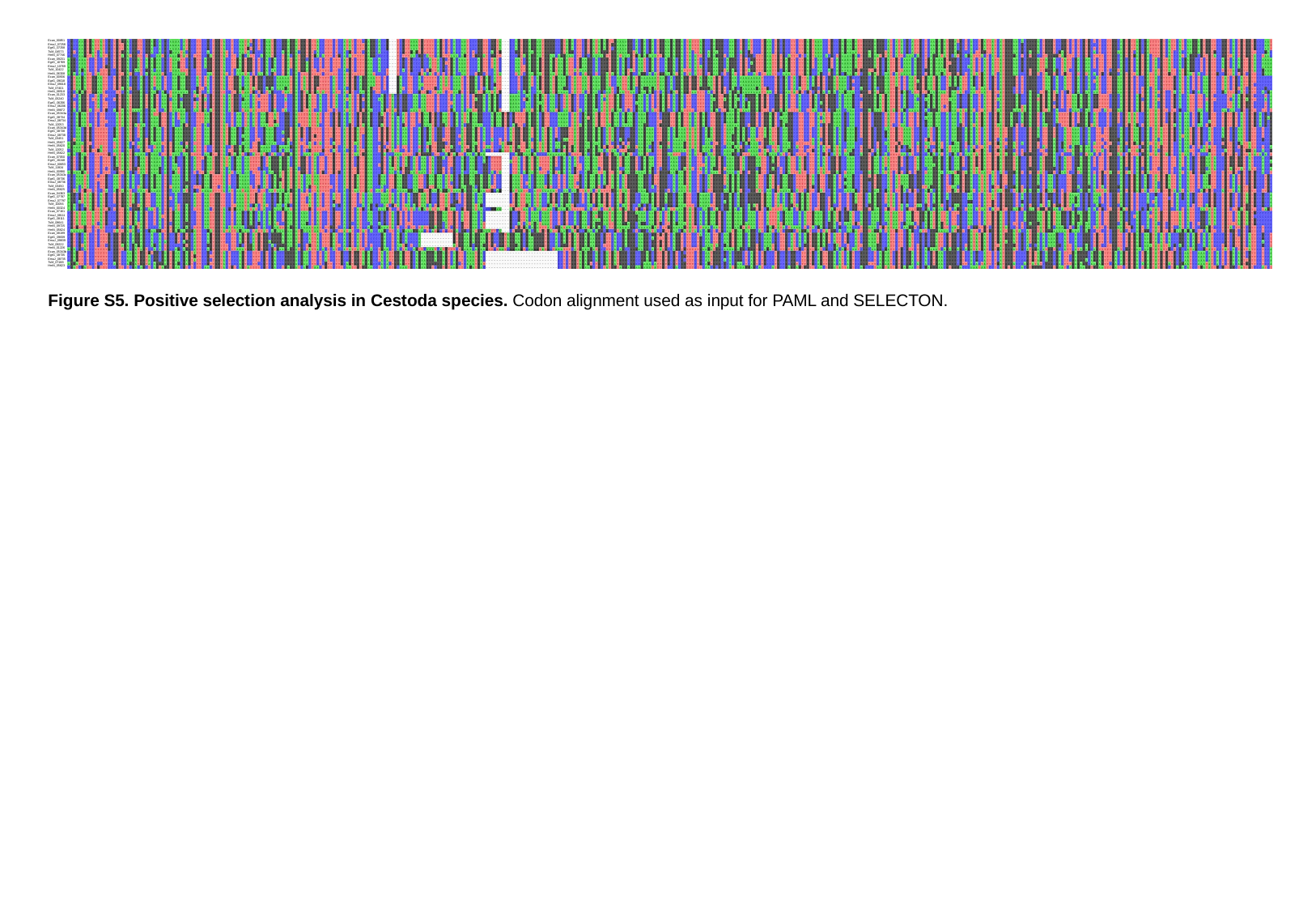

Ecan_00851
EmuJ_07258
EgrG_07258
TsM_04573
HmN_07746
Ecan_09231
EgrG_10769
EmuJ_10769
TsM_10422
HmN_06308
Ecan_00936
EgrG_09018
EmuJ_09018
TsM_07401
HmN_08518
Ecan_01233
TsM_05240
EgrG_06206
EmuJ_06206
HmN_09872
Ecan_05343a
EgrG_08734
EmuJ_08734
TsM_12053
Ecan_05343d
EgrG_08738
EmuJ_08738
TsM_03451
HmN_05627
HmN_05620
TsM_12052
HmN_05622
Ecan_07050
EgrG_20248
EmuJ_00653
TsM_11924
HmN_00990
Ecan_05343c
EgrG_08736
EmuJ_08736
TsM_03450
HmN_05625
Ecan_04363
EgrG_07797
EmuJ_07797
TsM_10056
HmN_00324
Ecan_07161
EmuJ_08111
EgrG_08111
TsM_08641
HmN_09725
HmN_05624
Ecan_08199
EgrG_09839
EmuJ_09839
TsM_09222
HmN_01226
Ecan_05343b
EgrG_08735
EmuJ_08735
TsM_07188
HmN_05623
Figure S5. Positive selection analysis in Cestoda species. Codon alignment used as input for PAML and SELECTON.
